# Revolutionizing goat milk gels: A central composite design approach for synthesizing ascorbic acid-functionalized iron oxide nanoparticles decorated alginate-chitosan nanoparticles fortified smart gels

**DOI:** 10.1016/j.heliyon.2023.e19890

**Published:** 2023-09-06

**Authors:** Shweta Rathee, Ankur Ojha, Kshitij RB. Singh, Vinkel Kumar Arora, Pramod Kumar Prabhakar, Shekhar Agnihotri, Komal Chauhan, Jay Singh, Shruti Shukla

**Affiliations:** a Department of Food Science and Technology, National Institute of Food Science Technology Entrepreneurship and Management, Kundli, Sonipat, India; b Department of Chemistry, Banaras Hindu University, Varanasi, Uttar Pradesh, India; c Department of Food Engineering, National Institute of Food Science Technology Entrepreneurship and Management, Kundli, Sonipat, India; d Department of Agriculture and Environment Sciences, National Institute of Food Science Technology Entrepreneurship and Management, Kundli, Sonipat, India; e Department of Nanotechnology, North-Eastern Hill University (NEHU), Shillong, Meghalaya, India

**Keywords:** Goat milk gel, Complex modulus, Flow stress, Extrudability, Ascorbic acid, Nanoparticles, Security

## Abstract

Goat milk gels (GMGs) are popular food due to their high water content, low-calorie density, appealing taste, texture enhancers, stability, and satiety-enhancing characteristics, making them ideal for achieving food security and zero hunger. The GMGs were optimized using the central composite design matrix of response surface methodology using goat milk powder (35–55 g), whole milk powder (10–25 g), and potato powder (10–15 g) as independent variables. In contrast, complex modulus, flow stress, and forward extrudability were chosen as dependent variables. The maximum value of complex modulus 33670.9 N, good flow stress 7863.6 N, and good extrudability 65.32 N was achieved under optimal conditions. The optimized goat milk gel was fortified with ascorbic acid-coated iron oxide nanoparticle (magnetic nature) decorated alginate-chitosan nanoparticles (AA-MNP@CANPs), making it nutritionally rich in an economically feasible way—the decorated AA-MNP@CANPs characterized for size, shape, crystallinity, surface charge, and optical characteristics. Finally, the optimized fortified smart GMGs were further characterized *via* Scanning electron microscopy, Rheology, Texture profile analysis, Fourier transforms infrared (FTIR), and X-Ray Diffraction (XRD). The fortified smart GMGs carry more nutritional diversity, targeted iron delivery, and the fundamental sustainability development goal of food security.

## Highlights

• Central composite design is a predictive regression modelling economic approach.
• Goat milk gels optimized using central composite design approach.
• Optimized Goat milk gels fortified with ascorbic acid-coated iron oxide nanoparticle decorated alginate-chitosan nanoparticles for smart gels.
• Nanofortification-enhanced goat milk gels for optimum health.

## Graphical abstract

**Figure:**
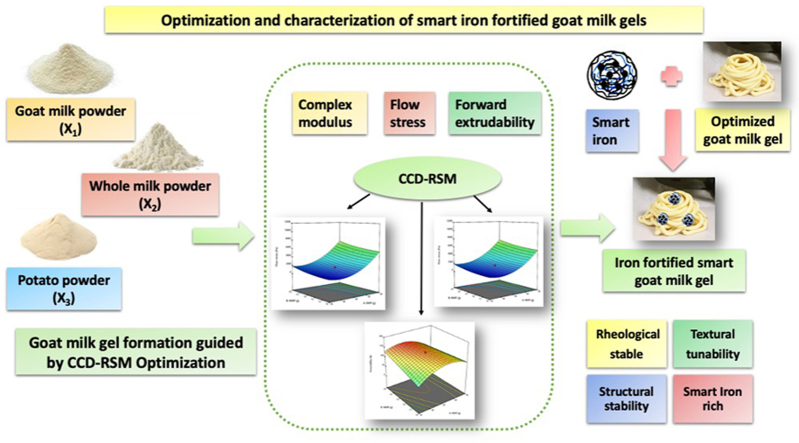

## 1 Introduction

Goat milk and its products first got attention in the United States in the 1960s because of its nutraceutical characteristics such as the similarity to human milk, soft curd formation, small milk fat globules, high β-casein content, high nutritional value, lower α−S1-casein, and low sugar content [1]. Among different products, goat milk gels (GMGs) are becoming popular due to their high water content, low-calorie density, appealing taste, texture enhancers, stability, and satiety-enhancing characteristics. They form through aggregation, phase separation, and self-assembling proteins/colloids in suspensions into ubiquitous weakly elastic solids [2,3]. They are biocompatible, eco-sustainable, and have tunable mechanical, microstructural, textural, and rheological characteristics [[4], [5], [6]]. The microstructure, texture, and rheology interconnection help to achieve smart rheology and mechanics that can be uniquely tuned for bioactive compounds' controlled release, texture, and design, designing complex shape foods, especially in 3D and 4D printability, making them ubiquitous in personal care, foods, and biomedical engineering [7,8]. In 2012, EFSA (European Food Safety Association) reported goat milk protein as a suitable protein source in infants and follow-on formulas [9]. According to the Grandview research, companies that are offering goat milk products through the online-retail channel are Supermarket Grocery Supplies Pvt Ltd (Big Basket), Aadvik Foods and Products Pvt. Ltd, Courtyard Farms, Farm Fresh, Nutragoat, Amazon, Flipkart, Wellversed, Cora Health & Wellness, and Vistara Farms Private Limited. The global market size was 12.45 billion USD in 2022 and is expected to grow at compounded annual growth rate (CAGR) of 4.7% from 2023 to 2030. The growing lactose intolerant population drives the growth, increasing number of health-conscious consumers, rising infant population, and technological developments [10]. Similarly, whole milk powder (WMP) offers good solubility, flowability, and rich and creamy flavor for developing GMGs. Potato powder (PP) is a potent gel-making material because its low gelatinization temperature gives excellent binding power, high consistency on pasting, white color, low gluten, and good transparency to the GMGs [11].

**Fig. 1:**
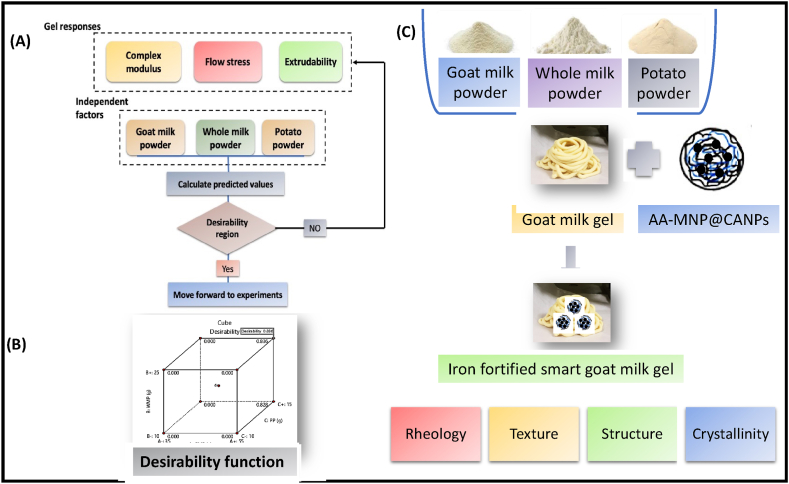
(A) Proposed flowchart demonstrated the potential application of CCD-RSM for goat milk gel formation with good complex modulus, flow stress, and extrudability (B) Desirability function (C) Fortified smart goat milk gel formation and its characterization.

Despite the many benefits of goat milk, there are significantly fewer products available in the market, so we used the central composite design of response surface methodology (CCD-RSM) for optimizing the GMGs with the best rheological and textural characteristics [12]. In a previous study, Patruni et al. [13] used RSM to optimize the process conditions for developing mixed gels using aloe vera and high methoxy pectin. They also compared the RSM results with artificial neural network (ANN) prediction and concluded that RSM could provide the optimal solution similar to ANN. Inspired by this, CCD-RSM could be an ideal approach for optimizing GMGs formation. The GMGs are novel biphasic systems with different polarity phases as they possess the remarkable rheological properties of polysaccharides and good interfacial properties of proteins with more strength than pure gels. In addition, the structural and physicochemical attributes, such as ligand binding, surface activity, self-assembly, gelation, environment-responsive behavior, and electric characteristics, make them reliable [14]. Modeling GMGs formation is complex due to the different constituents of polymers [15]. The characteristic responses, such as complex modulus, flow stress, and forward extrudability, are chosen using the CCD, as shown by the flowchart demonstration in Fig. 1A and selected using the desirability function depeicted in Fig. 1B. Optimized GMGs are an excellent source of nutrition with good rheological and texture characteristics but are iron deficient. So we used the fortification approach with earlier developed ascorbic acid-functionalized iron oxide nanoparticles (MNPs) decorated alginate chitosan nanoparticles (AA-MNP@CANPs) by our team as shown in Fig. 1C, to carry more nutritional agents to improve nutritional diversity and targeted iron delivery [16]; this is a fundamental goal for sustainable food security enable to achieve zero hunger sustainability development goal 2030 [17]. The fortified smart GMGs provide a sustained iron release and are dual stimuli-responsive to pH and magnetic field. There has been literature reported on different food gels. However, no one has studied AA-MNP@CANPs fortified smart GMGs.

We present a strategy that stands to solve multifaceted problems. The objective of this research aims to (I) model the effects of WMP, GMP, and PP for enhancing GMGs' rheological and textural parameters, (II) fortify the optimized GMGs with AA-MNP@CANPs to make stimuli-responsive and iron-fortified and (III) characterization of fortified GMGs using the rheology, texture, Infrared microscopy, SEM, and XRD. This work anticipates a facile class of AA-MNP@CANPs fortified smart GMGs that are malleable to various food, pharmaceutical, and industrial applications for achieving global food security.

## 2 Material and methods

### 2.1 Materials

The goat milk powder (27% protein, 36% carbohydrates, 28.5% Fat, 3% moisture) provided by (aadvik foods, India) and whole milk powder (34% protein, 38% carbohydrates, 26.2% fat, 3.37% moisture) from (Puramio foods, India). The potato powder (39.8% carbohydrates, 9.4% fat) was purchased from (Aekm Foods, India). Sodium alginate ((C_6_H_7_NaO_6_)n, analytical grade), chitosan (C_12_H_24_N_2_O_9_, Degree of acetylation> 75%, analytical grade), ferrous sulphate (FeSO_4_.7H_2_O, Purity> 99%), ferric chloride (FeCl_3_.6H_2_O, Purity≥ 99%), calcium chloride (CaCl_2_.6H_2_O, Purity∼98%) and l-ascorbic acid (C_6_H_8_O_6,_ Purity∼ 99%) were obtained from (Sigma-Aldrich, USA).

### 2.2 Formulation of goat milk gels

At room temperature, the specified amount of GMP, WMP, and PP was mixed as per the chosen responses shown in Table 1 and the design of 20 experimental runs depicted in Table 2. The prepared mixture was homogenized at 8000 rpm (Ultra-Turrax, T 25, IkaWerke, Germany) for 3 min for complete dissolution of powders in 50 mL. The obtained mixture was then heated in a 90 °C water bath for 10 min to develop GMG [18]. A similar procedure was used for all the 20 experimental runs defined combinations. After the GMG formation was stored at 4 °C for complete rehydration and gelation. Further, the prepared GMG was brought to ambient conditions immediately for following rheological and texture profile analysis.

**Table 1:** Independent factors and levels of CCD for the preparation of GMGs.

Independent factors	Levels					Responses
	-α	−1(low)	0(centre)	+1(high)	+α	
**X**_**1**_	28.18	35	45	55	61.82	Complex modulus
**X**_**2**_	4.89	10	17.5	25	30.11	Flow stress
**X**_**3**_	8.30	10	12.5	15	16.70	Extrudability

**Table 2:** CCD-RSM for modeling the effects of ingredient factors on complex modulus, flow stress, and forward extrudability of GMGs.

Runs	Factors/Ingredients (X)			Response Variables (Y)		
	GMP (g/g) (X_1_)	WMP (g/g) (X_2_)	PP (g/g) (X_3_)	Complex modulus (Pa) (Y_1_)	Flow stress (Pa) (Y_2_)	Extrudability (N) (Y_3_)
1	61.8	17.5	12.5	37324.5	11110.5	18.32
2	35	25	15	19301.4	2640	84.42
3	35	10	15	8757.5	408	60.7
4	55	25	15	33630.1	7850	65.03
5	45	30	12.5	23371.4	3665	152.7
6	28	17.5	12.5	14790.5	2560	24.24
7	45	4.9	12.5	16577.5	1420	113.8
8	45	17.5	12.5	9017.6	1650.8	138.26
9	45	17.5	8.3	3450.3	403	24.09
10	45	17.5	12.5	9000.8	1647	137
11	45	17.5	12.5	9080.8	1620.4	136.5
12	35	10	10	10291.3	650	14.04
13	45	17.5	16.70	13259.6	1795	81.5
14	55	25	10	20250.7	5930	37.8
15	55	10	10	22700.5	5485	24.09
16	45	17.5	12.5	8912.6	1652.7	137.8
17	55	10	15	24381.6	5180	121.5
18	45	17.5	12.5	9010.5	1659	136.2
19	35	25	10	9100.8	640	112
20	45	17.5	12.5	9013.8	1610	136.2
O	55	25	15	33670.89	7863.6	65.32
E	55	25	15	32718.52	7580.2	70.35

**GMP:** Goat milk powder**, WMP:** whole milk powder**, PP:** potato powder**.**

### 2.3 Goat milk gel characterizations

The dynamic oscillatory properties of GMGs were performed on a rheometer (MCR 52, Anton Paar, Austria) using 50 mm parallel plate steel geometry with a measuring gap of 1 mm. The excess gel was removed using a spatula. The dynamic oscillatory strain amplitude sweep measurement was performed at a frequency of 6.28 Hz by varying the strain from 0.01 to 100%. In these studies, storage modulus (G′) and loss modulus (G″) was used to determine the viscoelastic characteristics. The complex modulus G*, the overall resistance to material deformation, is a suitable indicator for gel viscoelastic behavior. The complex modulus was calculated as per equation (1) [19]. The storage (G′) and loss (G″) modules defined the elastic and viscous behavior of the gel and reflected its mechanical properties. The G′ values were significantly higher than those of G″, indicating that the formulation had gel-like structures dominated by elasticity, a feature that maintained its shape [14,15], confirming its viscoelastic nature [20]. Flow stress, the point at which the G’ crosses the G”, is an essential rheological parameter for gel fabrication. It also indicated the extrudability of the material, which implies the force necessary for extrusion [21].

(1) $$G^{*}=\sqrt[{2}]{G^{\prime 2}+G^{\prime \prime 2}}$$

The force necessary to extrude the material for assessing its rheological characteristics is forward extrudability. It was performed with the forward extrusion probe (HDP/FE) of the Texture profile analyzer (TA-HD PlusC, Stable Microsystems Ltd. UK) containing 100 g of sample loaded in a polycarbonate cylindrical container (100 mm × 50 mm; height × diameter) and 5 mm diameter base disk. The probe penetration was up to 20 mm at a test speed of 1.0 mm/s and then pulled out at a post-test rate of 10 mm/s [22].

### 2.4 Experimental design for goat milk gel forming parameters using CCD-RSM

This research work used CCD-RSM to find the optimum amount of GMP, WMP, and PP in preparing GMGs with excellent rheological and textural characteristics. The rheological properties can be best explained in terms of complex modulus and flow stress. As the complex modulus defines, the overall viscoelastic behavior of GMGs and flow stress is also crucial for the height of the structure maintained for the products developed. The extrudability is chosen for analyzing the textural property, as GMGs need to be squeezed for the product's commercial use. A preliminary screening study was done to find GMGs' complex modulus, flow stress, and extrudability. The ranges for the amount of GMP, WMP, and PP were chosen to develop GMGs [8]. Three independent variables were selected from as **X**_**1**_: GMP (g/g), **X**_**2**_: WMP(g/g), and **X**_**3**_: PP(g/g) and coded at five levels as –α, −1, 0, +1, and +α, in which the number of α is (2)^K/4^ = 1.676, where k is the number of independent variables. The complex modulus, flow stress, and extrudability were selected as responses of the GMG denoted as (Y). The actual and coded values of the independent variables are displayed in Table 1. According to the CCD matrix, the number of 20 runs was come out using the statistical package (Design expert13, Stat Ease Inc., MN, USA). The runs are calculated as (2^k^+ 2 k + cp), where k is the independent variable and cp is the central point. The experiment incorporating eight factorial points, six axial points, and six replicated center points was proposed, as shown in Table 2. The response variable from the suggested experimental runs is expressed as a second-order polynomial regression equation (2) given below. In addition, model reliability is determined by residual standard errors (RSE).

(2) $$Y=\beta_{0}+\sum_{i=1}^{n}B_{i}X_{i}+\sum_{i=1}^{n}B_{\text{ii}}{X_{i}}^{2}+\sum_{i=1}^{n-1}\sum_{j>1}^{n}\beta_{\text{ij}}\;X_{i}X_{j.}+\varepsilon$$

where *Y* represents the predicted responses, *Xi*, *X2i,* and *Xj* represent the levels of independent variables in coded levels, *βi, βii, and βij are coefficients for linear, quadratic, and interaction effects, respectively* β_0_ is model coefficient, *n is the number of factors and ε* is the model's error. CCD-RSM was used to model the impact of independent variables by mathematical models fitting and selecting factor levels by optimizing the response employed per the Design Expert 13 software. The model significance, model fitting, and adequacy were demonstrated by correlation coefficient (R^2^), Fisher's variation ratio (F-value), probability value (Prob > F), adequate, and coefficient of variation % (CV). The lower value of CV < 10 for all three responses indicated the reproducibility and reliability of the data. The 2D contour plots, 3D response surface plots, perturbation plots, histogram plots, and box plots express the fitted model equations [23]. The optimized conditions were determined to obtain a maximum response of GMP, PP, complex modulus, flow stress, and extrudability, and the levels of WMP were set in range.

### 2.5 Development of AA-MNP@CANPs fortified smart goat milk gel

The synthesis of AA-MNPs is done in two steps. Initially, the co-precipitation of Fe (II) and Fe (III) salt in an alkaline medium, followed by the coating of AA by hydrothermal treatment in a single step. The CANPs nanoparticles are prepared by ionotropic gelation and used to decorate AA-MNPs since they offer a large surface area, biocompatible, eco-friendly, and easy to prepare. The AG: CS mass ratio of 5:1 and AG concentration were optimized for CANPs preparation. The developed AA-MNP@CANPs are characterized by colloidal stability by particle size and zeta potential analysis. Additionally, their structural analysis by TEM imaging. Finally, spectral analysis by UV–visible spectroscopy and FT-IR can be seen in (Section 2 of supplementary information). The 8 mg of freeze-dried AA-MNP@CANPs were added to the optimized GMGs with continuous shaking in the water bath for 5 min at 45 °C to develop fortified smart GMGs [24]. The developed fortified smart GMGs were stored at 4 °C overnight for complete rehydration and gelation [25]. The fortified smart GMGs were used for rheological, spectroscopic analysis, gel particle morphology, and instrumental texture profile analysis.

### 2.6 Characterization of fortified smart goat milk gels

#### 2.6.1 Dynamic rheology

For oscillatory shear measurements, gels were given sinusoidal strain with amplitude varying from 0.01 to 100% at a frequency of 6.28 Hz to estimate the gel's linear viscoelastic region (LVE). After determining the LVE, dynamic oscillatory frequency sweep measurements were performed within 1% strain amplitude, angular frequency 1–100 rads^−1^ [26]. The 3 Interval Thixotropy Test (3ITT) rotational recovery behavior assessed the molecular structure rebuilding after a shear disruption by simulating the gels' extrusion process and shape leveling. Initially, 1 s^−1^ shearing was given for 180 s, followed by 100 s^−1^ for 120 s to model the extrusion process. Finally, 1 s^−1^ shearing for 300 s to pattern the gel state after extrusion. The recovery rate (*RR*) was analyzed by the viscosity ratio in the third and the viscosity in the first stages. Finally, a dynamic temperature sweep was performed to check the material's behavior at the heating ramp from 25 °C to 60 °C at a temperature ramp of 3 °C/min.

#### 2.6.2 Spectroscopic analysis

The nature of compounds is checked by estimating the functional groups and bonds involved. The Fourier transformed infrared spectroscopy (FT-IR) was used in the spectral range from 550 to 4000 cm^−1^ on FTIR spectrophotometer (Nicolet iS5, Thermo Fisher Scientific) in the spectral range of 400–4000 cm^−1,^ and measurements were recorded in percent transmission (%T). Samples were freeze-dried for analysis. The X-ray diffraction (XRD) was performed to examine the crystalline nature of the gels on the X-ray diffractometer (D8 Advance, Bruker) with Cu-Kα radiation (*λ* = 1.5406 Å) in the 2*θ* diffraction angles ranging from 10° to 80°.

#### 2.6.3 Gel particle morphology

After the optimization study, the shape and topography of synthesized smart GMGs were monitored using scanning electron microscopy after freeze-drying samples using an EVO18 system (Zeiss, Germany) for analyzing the particle morphology.

#### 2.6.4 Instrumental texture profile analysis (TPA)

The Textural Profile Analysis (double compression) test used a TA.XT plus Stable Micro Systems Texture Analyser (Stable Microsystems Ltd., Surrey, England) with a flat, circular compression plate (75 mm diameter, P/75). The compression speed of 5 mm/s up to a distance of 20 mm with a 10 g trigger force [27]. The Firmness, adhesiveness, springiness, and cohesiveness were recorded.

## 3 Results and discussion

### 3.1 Effect of variables on the characteristics of goat milk gels

CCD-RSM, a statistical modeling design, was used to optimize the effect of factors and their interaction to maximize the complex modulus (Y_1_), flow stress (Y_2_), and extrudability (Y_3_) of GMGs. According to CCD, 20 experimental runs were performed by data points as given in Table 2. The complex modulus for trial GMG formulations was found in the 14790–373324.5 Pa, flow stress range was between 408 and 11110.5 Pa while the extrudability was 14.04–152.7 N. The basis for selecting quadratic models for all the responses was the sequential sum of squares (F-value, *p*-value) and fit summary (adjusted and predicted R^2^). The quadratic model (highest order polynomial) was selected for all the responses as the model is not aliased, and additional terms are significant. According to the fit data summary, quadratic models were chosen to predict the complex modulus**,** flow stress**,** and extrudability on GMGs given in best model fit equations (3- 5). These models were developed after ignoring insignificant variables and were found to be significant with *p* < 0.05 and lack of fit value (>0.05) [28].

Complex modulus

(3) $$\left(G*\right)=9010.23+6693.30\;\text{GMP}+2019.37\;\text{WMP}+2945.38\text{PP}-319.34\;\text{GMP}*\text{WMP}+799.21\;\text{GMP}*\;\text{PP}+2929.09\;\text{WMP}*\;\text{PP}+6001.05\;{\text{GMP}}^{2}+3850.36\;{\text{WMP}}^{2}-257.75\;{\text{PP}}^{2}$$

Flow Stress

(4) $$=1640.20+2525.26\;\text{GMP}+667.26\;\text{WMP}+418.40\;\text{PP}-11.63\;\text{GMP}*\text{WMP}-17.87\;\text{GMP}*\;\text{PP}+558.38\;\text{WMP}*\;\text{PP}+1835.38\;{\text{GMP}}^{2}+317.67\;{\text{WMP}}^{2}\;–\;192.69\;{\text{PP}}^{2}$$

Extrudability

(5) $$=136.99-2.39X_{1}+10.57\;\text{WMP}+17.59\;\text{PP}-20.55\;\text{GMP}*\text{WMP}+13.19\;\text{GMP}*\;\text{PP}-18.05\;\text{WMP}*\;\text{PP}-40.92\;{\text{GMP}}^{2}-1.33\;{\text{WMP}}^{2}\;–\;29.78\;{\text{PP}}^{2}$$

The complex modulus (Y_1_) describes the entire viscoelastic behavior of GMGs since it's a combination of both storage modulus/G′ and loss modulus/G” [29]. It varied between 14790.5 and 37324.5 Pa and increased with GMP increase, followed by WMP and PP. Using the mathematical modeling approach as shown in equation (3)**,** linear terms GMP and PP positively increased the complex modulus of GMGs from 14790 to 373324.5 Pa. The maximum value of complex modulus 373324.5 Pa was obtained at maximum GMP 61.82 g and intermediate 12.5 g PP. This phenomenon is described due to the stronger protein aggregation induced by GMP's higher casein content in a gel system [30]. The GMP further enhanced the gel viscoelasticity by strengthening the disulfide bonds or hydrogen bonding with PP acted as a filler. Researchers additionally observed that the high-fat content in GMP also improved the viscoelastic behavior of gels [31]. The previous studies by different research groups also observed similar results that an increase in milk powder increased the viscoelastic behavior of gels [32,33]. The root effect of the interrelationship between the chosen factors and individual responses expressed by the quadratic model and respective statistical significance was determined by ANOVA from Table 3. The F-test value with a low probability score (*p* < 0.0001) indicated the high statistical significance of the model in depicting the actual relationship between the obtained data and response variables. In addition, the determination coefficient R2 (0.9999) value indicates that 99.99% of the variation is connected to the three independent variables. The predicted R2 (0.9977) measures the regression of goodness of fit for the model, and it's in reasonable agreement with the adjusted R2 (0.9998) with less than 0.2 difference. The lower CV value is 0.56% indicates the reproducibility and reliability of the data [34].

**Table 3:** ANOVA table for a quadratic model for complex modulus, flow stress, and extrudability.

Source	Complex modulus		Flow stress		Extrudability	
	F-value	Prob > F-value	F-value	Prob > F-value	F-value	Prob > F-value
**Model**	22200.59	<0.0001	32599.95	<0.0001	2396.48	<0.0001
**GMP**	78715.04	<0.0001	1.713E+05	<0.0001	35.66	0.0001
**WMP**	7164.85	<0.0001	11959.55	<0.0001	695.05	<0.0001
**PP**	15242.62	<0.0001	4702.37	<0.0001	1925.91	<0.0001
GMP*WMP	104.96	<0.0001	196.06	<0.0001	1539.95	<0.0001
GMP* PP	657.41	<0.0001	5.03	0.0488	634.58	<0.0001
WMP* PP	8830.45	<0.0001	4905.92	<0.0001	1187.81	<0.0001
**GMP**^**2**^	66770.28	<0.0001	95484.90	<0.0001	10993.92	<0.0001
**WMP**^**2**^	27487.24	<0.0001	2860.39	<0.0001	11.66	0.0066
**PP**^**2**^	123.18	<0.0001	1052.42	<0.0001	5821.90	<0.0001
**Lack of Fit**	4.34	0.0642	1.58	0.3144	4.84	0.0542
Model statistics						
**CV%**	0.5666		0.7969		1.69	
**R**^**2**^	0.9999		1.0000		0.9995	
**Adj-R**^**2**^	0.9998		0.9999		0.9991	
**Predicted R**^**2**^	0.9977		0.9998		0.9969	

GMP: Goat milk owder, WMP: Whole milk powder, PP: Potato powder, CV%: Coefficient of variation, R^2^: Coefficient of determination, Adj-R^2^: adjusted coefficient of determination.

**Fig. 2:**
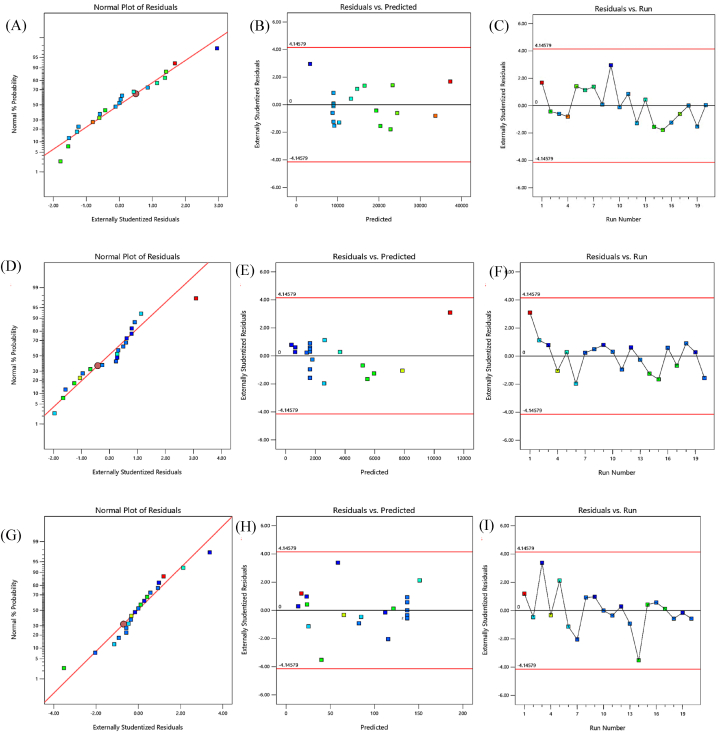
Diagnostic plots for complex modulus (Upper panel), flow stress (middle panel), and extrudability (lower panel) on GMGs: **(A)(D)(G)** normal probability plots on probit scale, (B)(E)(H) externally residuals versus predicted values plots, **(C)(F)(I)** externally residual versus run number plots.

Meanwhile, lack of fit is an evaluation of discrepancies between actual and predicted values, having a value of 4.34 higher than 0.05, showing an insignificant effect. For analyzing the complex modulus effect, Fig. 2A showed the normal % probability plot versus externally residuals falling in a straight line, which indicates that the errors are normally distributed. In addition, Fig. 2B illustrates the externally residuals versus predicted values plot, showing the horizontal band around the residual = 0, indicating the adequacy of the model. Further, Fig. 2C shows the plot of externally residuals versus the run order of complex modulus, highlighting the residuals' distribution along the run number. The flow stress/yield point (Y_2_) is defined as the crossover point of storage modulus and loss modulus in a stress sweep measurement and the estimated stress at collapse directly proportional to object height for commercial food materials such as tomato puree, mayonnaise, meat, and vegetable-based spreads [35]. It varied between 408 and 11110.5 Pa in GMGs [36] and increased significantly with the increase in GMP than PP. The mathematical model given in equation (4) showed that all the model terms synagonistically affected the flow stress except interaction terms GMP*PP and quadratic term **PP**^**2**^**.** The maximum value of flow stress 11110.5 Pa was obtained at the maximum GMP 61.82 g and intermediate amo unt 12.5 g of PP. The authors attributed this finding to the mutualistic effect of PP-calcium-casein interactions after the dissociation of micellar casein [37]. The presence of PP excluded the water molecules from proteins, reduced mobility, and increased the flow stress of gels [38]. Apart from it, several other factors, such as casein micelle size, casein proportions, mineral content, and complex interactions, might affect the flow stress. The GMP has provided more structural strength due to the crosslinking between the casein and starch molecules [39]. The research team led by Lee et al., 2020 also observed an increase in flow stress due to the increase in milk powder due to the enhanced colloidal network collisions in 3D printing milk-based products [40]. Similarly, Lille et al., 2020 studied the effect of milk powder and rye flour on 3D-printed snacks. They observed the same increase in the flow stress with increasing the amount of milk powder and wholegrain rye flour [41]. The ANOVA in Table 3 showed that flow stress was significantly affected by linear, quadratic, and interaction terms with *p* < 0.05. The high value of the R^2^ (1.000) detonated that the quadratic model was highly significant. The adjusted R^2^ (0.9999) is in reasonable agreement with the predicted R^2^ (0.9988), indicating the model's goodness of fit. The lower CV% value was 0.7969, demonstrating the quadratic model reliability. The insignificant lack of fit with a value of 1.58 > 0.05 indicated that this model has good predictability. The scattering of values in Fig. 2D for the normal % probability plot versus externally residuals showed the model's fitness for the flow stress. It also showed that the flow stress values were located in a narrow range of normal probability lines, diagonal lines, and two zero axes, suggesting the accuracy of the predicted model for depicting the correlation between the variables and responses. Similarly, in Fig. 2E, the externally residual versus predicted plot for flow stress showed the scattered predicted values on the x-axis around 0 on the y-axis, confirming the goodness of fit. In addition, the influence of run orders on the design was shown in Fig. 2F by model externally residuals versus run orders, indicating the non-dependence of errors.

The extrudability (Y_3_) measures the compressive force required to flow the gel from the nozzle. It's a critical textural property necessary for selecting packing material and removing the gel from the container [42]. It varied between 14.04 and 152.7 N and positively correlated with PP and WMP. The regression model equation for studying the effect of model terms on extrusion assay equation (5) showed that only three terms positively increased the extrudability. The maximum value of extrudability 152.7 N was obtained at 12.5 g of PP and 30 g of WMP. The increase in PP was more prominent due to potato starch's toughness and higher strength [43]. Although PP is used in fewer amounts, the presence of starch-associated proteins enhanced the extrudability effect of GMGs [44]. For instance, a research team led by Martinez-Monzo et al., 2019 observed an increase in the extrudability of potato puree formulation by increasing the amount of potato powder [45]. This was mainly due to the increased intermolecular hydrogen bonding resulting in the compact.

Network structure and gel strength [46]. Another supported study by Zhu et al., 2021 observed the peroxidase-treated potato flours with more protein interactions and a more homogeneous gel network [47]. The GMP has a negative effect on the extrudability of gels due to the lubricating effect of GMP's high-fat content than the WMP and PP [48]. The model with an F-value of 23964.8, as demonstrated in ANOVA Table 3, with *p* < 0.05 considered significant. The difference between adjusted R^2^ value (0.9991), and the predicted R^2^ (0.9969) was less than 0.2, indicating the model's good prediction. The insignificant lack of fit value 4.84 > 0.05 demonstrated good model predictability. Further, the model reproducibility was confirmed with a low CV% value (1.69) <10%. In the current study, Fig. 2G, the normal % probability versus externally residuals showed a straight line distribution, indicating the selected model acceptability. Fig. 2H represents the externally residual versus the predicted plot with values aligned around 0 value. Further, Fig. 2I described the randomly scattered runs versus externally residuals plot, confirming the model adequacy.

**Fig. 3:**
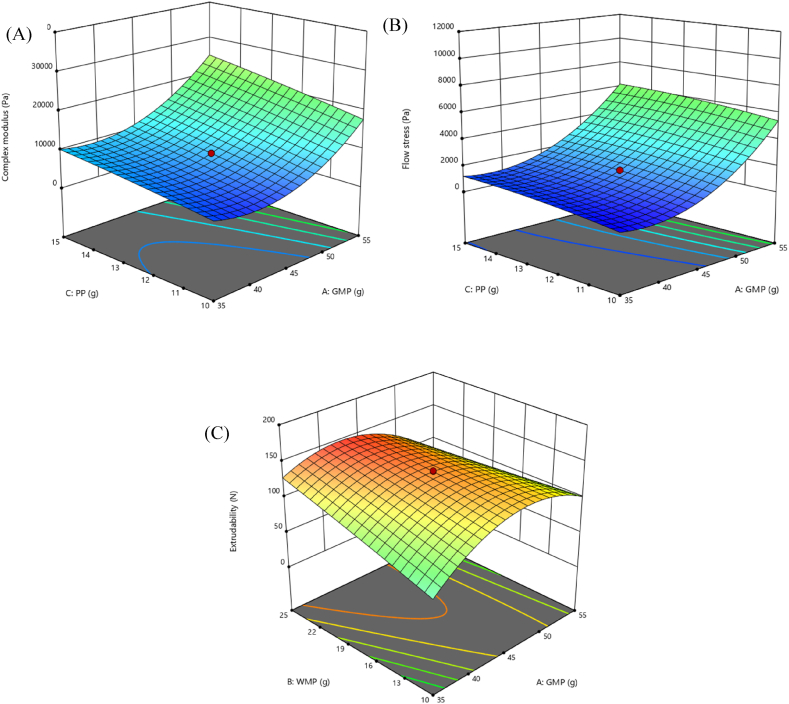
3D response surface plots for assessing the mutual effect of GMP and PP on **(A)** complex modulus, **(B)** flow stress, and GMP and WMP effect on **(C)** extrudability of GMGs.

The 3D response surface plots shown in Fig. 3 indicated the interaction effect of selected responses with the variables, which confirms the variable's effect. In Fig. 3A response plots showed more sensitivity of responses and the positive correlation relationship of GMP on complex modulus. Similar positive effect of GMP was observed on the flow stress as shown in Fig. 3B.

But the effect of the GMP was negative on the extrudability as depicted in Fig. 3C. In conclusion, the complex modulus, flow stress, and extrudability affected dependent variables, with GMP being the most important factor. In addition, as the GMP increases, the complex modulus and flow stress increase, and the extrudability decreases.

### 3.2 Models’ optimization and validation

The present work aimed to predict the effect of GMP, WMP, and PP on a GMG (G'>G″) with enhanced complex modulus, flow stress, and extrudability. For this purpose, GMP and PP amounts were maximized, and the WMP amount was set in range to develop the GMGs. The highest desirability of 0.83 was selected with the optimum ratio of ingredients for GMG development was GMP:WMP: PP = 55:25:15. During goal maximizing, the amount of ingredients favorable for optimizing the rheological and extrudability of GMGs were found to be 55 g GMP, 25 g WMP and 15 g PP. The experiment was conducted at the specified optimum amount of GMP:WMP: PP (55:25:15) to verify the validity of the CCD-RSM model results. The optimized complex modulus 33670.9 N agreed with the predicted complex modulus 32718.52 N. Similarly; the optimized flow stress 7863.61 N was higher than the predicted 7580.2 N. Finally, the optimized extrudability of 65.3207 N of optimized GMGs was less than the predicted 70.35 N. As a result, the model was verified because the experimental values were near the predicted values. Thus, the model helped predict the optimal amount for developing GMGs from GMP, WMP, and PP. The validation and optimization showed that the model's prediction agreed with the experimental results with less than 2% error, suggesting that the models were adequate and able to predict responses accurately.

### 3.3 Characterization of AA-MNP@CANPs fortified smart goat milk gels

The developed AA-MNP@CANPs are characterized by particle size and zeta potential Fig. S1. The structural analysis by TEM imaging is shown in Fig. S2—the spectral analysis by UV–visible spectroscopy and FT-IR Fig. S3. Finally, the developed AA-MNP@CANPs fortified smart GMGs are characterized in the section below using rheological, spectral, and microstructural analysis.

#### 3.3.1 Smart goat milk gel particle analysis

**Fig. 4:**
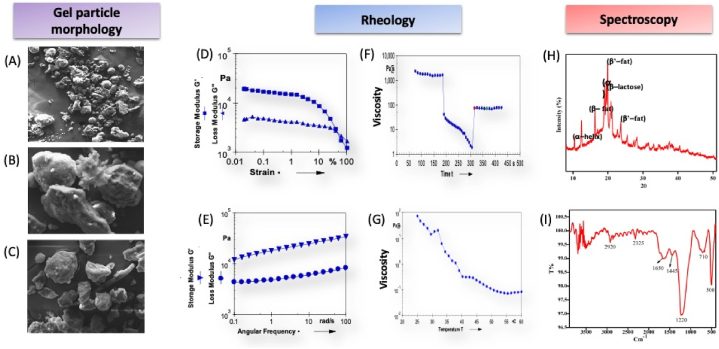
Gel particle morphology analysis using scanning electron microscope at scale bar 10–100 μm (A-10 μm) and (B, C-100 μm). Rheology analysis (D-amplitude sweep, E-frequency sweep, F-rotational recovery, and G-temperature sweep) and spectroscopy (H- X-ray diffraction pattern, I-Fourier transform infrared spectroscopy) of fortified smart goat milk gels.

The morphology of the smart GMG powder was examined by scanning electron microscope. The GMP content was more predominant than WMP, showing a complex network filled with PP in a network [49]. The proteins' of GMP and WMP regular aggregation occurred, forming hydrophobic bonds with lower bond energy that could hold part of the system as shown in Fig. 4A at a 10 μm scale. Similarly Fig. 4B and C show the gel micrograph at 100 μm scale.

#### 3.3.2 Dynamic rheology

The viscoelastic behavior of the GMGs was characterized by various rheological methods. For instance, Fig. 4D illustrates the strain sweep of GMGs to estimate the linear viscoelastic range (LVE**),** which can be used to evaluate the viscoelastic property of smart GMGs. The value of storage moduli is greater than loss moduli (G’>G″) within the LVE (0.1%–10%), followed by a crossover point where gel-sol transition occurs (G”>G′) and gel starts to flow. The large LVR observed due to the gel particle's proximity and deformation occur when large stress is applied [50]. Similar results were observed in earlier studies with increased hydrocolloid addition results in increased LVE range due to enhanced particle interaction [51]. Thus, the smart GMG exhibited elastic-dominated (gel-like) behavior within the LVE range. In addition, frequency sweep analysis was convenient for studying the deformation and flow of viscoelastic samples and classifying gels into strong or weak gels and viscous sols shown in Fig. 4E [52]. It helped to understand the mechanical reaction at small oscillation strains within the LVE range. GMGs formed by covalent bonds were usually frequency-independent with G’> G,” indicating gel-like behavior. This can be explained by the fact that gel particles are densely packed and have higher percolation showing negligible change with frequency. In contrast, those created by non-covalent interactions show frequency dependence [53]. Further, Fig. 4F illustrates the rotational recovery ability of smart GMGs analyzed using a time sweep. In the initial step, G′ and G″ were practically constant for the first 200 s, and the correlation between the storage and loss modulus of smart GMGs was reversed at a high strain for 100s in the second step (G’’ > G′), indicating the elastic structure was broken under the strain change. In the final step, it started recovering when the strain returned from 100 to 0.01%, showing its viscoelastic nature. Finally, the smart GMGs' thermal stability was shown in Fig. 4G by studying the change in viscosity versus temperature. The heating increased from 25-60 °C, which decreased the viscosity due to the increase in the molecular kinetic energy, thereby overcoming attractive intermolecular forces [54].

#### 3.3.3 Spectroscopic analysis

The XRD spectra of the fortified smart GMGs showed sharp crystalline peaks at 10.5°,12.5°, 14°, 17°, 19.5°, 20.9°, 21°, 28°, and 80° indicating the existence of crystalline peaks with long-range order. The 10° and 19.5° peaks correspond to milk protein's α-helix and β-sheet structure. The milk fat showed peaks around 17° and 28° corresponding to milk fat β and β′- polymorph crystal structure [55]. The lactose monohydrate showed diffraction peaks around 12.5° and 20°. β−lactose showed the peaks around 10.5°, 20.9° and 21° [56]. It illustrates that the molecular microstructures transform into a regulated arrangement after the gel formation through the aggregation between polysaccharide molecules and synergistic reaction [57] Fig. 4H.

The FTIR spectra showed 3350 cm^−1^ corresponding to υN-H stretching of amide A (protein). The adsorption peaks at 2920 cm^−1^ correspond to CH_2_ from fatty acids, and 2325 cm^−1^ corresponds to υC-H stretching, υC≡C, or υC≡N stretching of alkynes or nitriles of milk proteins [58]. The peaks at 1650 cm^−1^ correspond to the amide I region due to the υC=O bonds of proteins depending on their secondary structure. The peak between (1500–1600 cm^−1^) corresponds to the amide II region due to the deformation of the N–H bond, a C–N bond [59]. The bands around 1400 cm^−1^ are ascribed to δCH_2,_ and the region between (1200-850 cm^−1^) fingerprint print region of carbohydrates due to potato starch [60]. Further, since the GMG is fortified with AA-MNP@CANPs, the characteristic C=O stretching vibration at 1691 cm^−1^ and stretching vibration coupled with vibrations along with the conjugated system at 1525.03 cm^−1^ for the C=C, confirming the formation of AA-functionalized MNPs [61]. Finally, CANPs displayed characteristic peaks around 1550 cm^−1^ due to C=C stretching and 1408 cm^−1^ due to N–H stretching, confirming the development of fortified smart GMGs Fig. 4I.

#### 3.3.4 Instrumental texture profile analysis

The fortified smart GMGs were analyzed for their textural profile analysis**.** Hardness represents the GMG firmness, defined as the gel strength under the first compression cycle. The hardness value was 7.7 ± 0.263 N and the results agreed with the soft-cheese hardness observed by Bareen and its team, indicating that the fortification did not affect the hardness of the GMGs [8]. The smart GMG's hardness is due to the closer crosslinking behavior due to PP acting as filling material in the heat-induced polymerized protein backbone to form a compact gel network [62]. However, there might be other interactions, including hydrogen bonds and hydrophobic or covalent bonds, which can stabilize the developed protein–polysaccharide matrix [63]. Researchers observed that GMGs firmness also depends on the total solids content, casein content, and micelle structure [64]. Adhesiveness is the negative area for the first bite and is work required to overcome the attractive forces between gel and other materials' surface and was about −10.0484 ± 0.698 N. Its low value indicated the smooth surface of GMGs and was not sticky [65]. Hardness and adhesiveness were the most critical textural characteristics studied for fortified smart GMGs [66]. Finally, the results showed that the fortification did not change the textural attributes of GMGs.

## 4 Conclusion

This scientific study successfully used the specialized central composite design/response surface methodology (CCD-RSM) to create novel GMGs. The optimized GMGs formulation contains 55 g GMP, 25 g WMP and 15 g PP with improved complex modulus, flow stress, and forward extrudability, fulfilling the prerequisite using the desirability approach. The optimized GMG was evaluated for its rheological and textural characteristics analysis. All the obtained results were under the conditions for gel development. The validation and optimization based on the software prediction suggested that this model was reliable and could accurately predict the GMG attributes. This study shows that RSM is a feasible approach to optimize fabrication to achieve GMGs with good rheological and textural properties based on the role of GMP, WMP, and PP in GMGs development.

Further, the optimized GMGs were fortified with AA-MNPs@CANPs to make them nutritional and stimuli responsive. The resulting fortified smart GMGs showed no difference in the gels' textural, rheological, morphological, and crystalline nature, facilitating the development of GMGs for the goat milk industry. The fortified smart GMGs have the potential to innovative food, pharmaceutical, and cosmetics applications for achieving food security, a vital sustainability development goal in 2030. The study contributes to scientific knowledge and potentially revolutionizes the way GMGs are used and perceived.

## Funding

Rathee would like to thank 10.13039/501100001501UGC, Delhi, for providing fellowship 1586/(NET-Dec 2018) for pursuing a Ph.D.

## Author contribution statement

Shweta Rathee: Conceived and designed the experiments; Performed the experiments; Analyzed and interpreted the data; Contributed reagents, materials, analysis tools or data; Wrote the paper.

Ankur Ojha, Kshitij RB Singh, Vinkel Kumar Arora, Pramod Kumar, Shekhar Agnihotri, Komal Chauhan, Jay Singh, Shruti Shukla: Analyzed and interpreted the data.

## Data availability statement

Data will be made available on request.

## Declaration of competing interest

The authors declare that they have no known competing financial interests or personal relationships that could have appeared to influence the work reported in this paper.
