# Supplementary material for: Revolutionizing goat milk gels: A central composite design approach for synthesizing ascorbic acid-functionalized iron oxide nanoparticles decorated alginate-chitosan nanoparticles fortified smart gels
Source: Heliyon. 2023 Sep 6;9(9):e19890. doi: 10.1016/j.heliyon.2023.e19890 (PMC10559278; doi:10.1016/j.heliyon.2023.e19890)
Supplement: Multimedia component 1 [file mmc1.docx]

**Supporting Information**

**Revolutionizing Goat Milk Gels: A Central Composite Design Approach for Synthesizing Ascorbic Acid-Functionalized Iron Oxide Nanoparticles Decorated Alginate-Chitosan Nanoparticles Fortified Smart Gels**

Shweta Rathee^1^, Ankur Ojha^1*^, Kshitij RB Singh^2^, Vinkel Kumar Arora^3^, Pramod Kumar Prabhakar^1^, Shekhar Agnihotri^4^, Komal Chauhan^1^, Jay Singh^2^, Shruti Shukla^5*^

^1^ Department of Food Science and Technology, National Institute of Food Science Technology Entrepreneurship and Management, Kundli, Sonipat, India

^2^ Department of Chemistry, Banaras Hindu University, Varanasi, Uttar Pradesh, India

^3^ Department of Food Engineering, National Institute of Food Science Technology Entrepreneurship and Management, Kundli, Sonipat, India

^4^ Department of Agriculture and Environment Sciences, National Institute of Food Science Technology Entrepreneurship and Management, Kundli, Sonipat, India

^5^ Department of Nanotechnology, North-Eastern Hill University (NEHU), Shillong, Meghalaya, India.

**^*^Correspondence to:**

Dr. Ankur Ojha [(aojha.niftem@gmail.com)](mailto:(aojha.niftem@gmail.com))

Dr. Shruti Shukla (shrutishukla1983@gmail.com)

Table of Contents

[S1. Instruments S1](#_Toc134490001)

[S2. Characterization S1](#_Toc134490002)

[S2.1 Particle size and zeta potential S2](#_Toc134490003)

[S2.2 Transmission electron microscopy S2](#_Toc134490004)

[S2.3 UV-VIS spectroscopy S2](#_Toc134490005)

[S2.4 Fourier Transform infrared spectroscopy (FT-IR) S2](#_Toc134490006)

# **S1. Instruments**

The optical characteristics were analyzed using UV-visible spectrophotometer (Shimadzu 2600). DLS data were collected using a particle size analyzer (NanoZS Zetasizer, Malvern). FTIR data were recorded using a Fourier Transform Infrared Spectrometer (Make: Agilent; Model: Cary 660 series). The structural analysis was performed using a TEM instrument (JEOL 2100 apparatus, Germany). The equipments mentioned above used in various experiments is located in NABI, Mohali.

# **S2. Characterization**

## **S2.1 Particle size and zeta potential**

The hydrodynamic diameter of the synthesized nanomaterial was determined using a particle size analyzer (Zetasizer, Malvern, UK). However, zeta potential is measured to analyze the surface charge and stability of the CANPs and decorated AA-MNP@CANPs. The nanomaterials were diluted in deionized water, sonicated for 15 minutes, and then subjected to dynamic light scattering and zeta potential analysis.

## **S2.2 Transmission electron microscopy**

Transmission electron microscopy (TEM) analysis is performed to deduce the morphology and size of the structures. The sample suspensions in deionized water were poured on a copper grid (carbon coated), followed by the removal of extra solution from the grid after 2 minutes. The dried sample remained on the copper grid and was subjected to TEM analysis.

S1

## **S2.3 UV-VIS spectroscopy**

UV-visible spectroscopy confirms the synthesis of CANPs and decorated AA-MNP@CANPs nanomaterials. The UV-visible spectra of the synthesized nanomaterial solutions in deionized water were recorded in the range of 200-700 nm using a Shimadzu UV-2600 spectrophotometer. This helps in confirming their respective characteristic peaks.

## **S2.4 Fourier Transform infrared spectroscopy (FT-IR)**

The Fourier transform infrared spectroscopy technique is used to measure the vibrational frequencies of the chemical bonds involved to detect the absence or presence of various functional groups on the surface of nanomaterials. Freeze-dried samples were analyzed by attenuated total reflection (ATR) spectroscopy at the resolution of 4 cm-1 using an FTIR spectrophotometer from Agilent technologies.


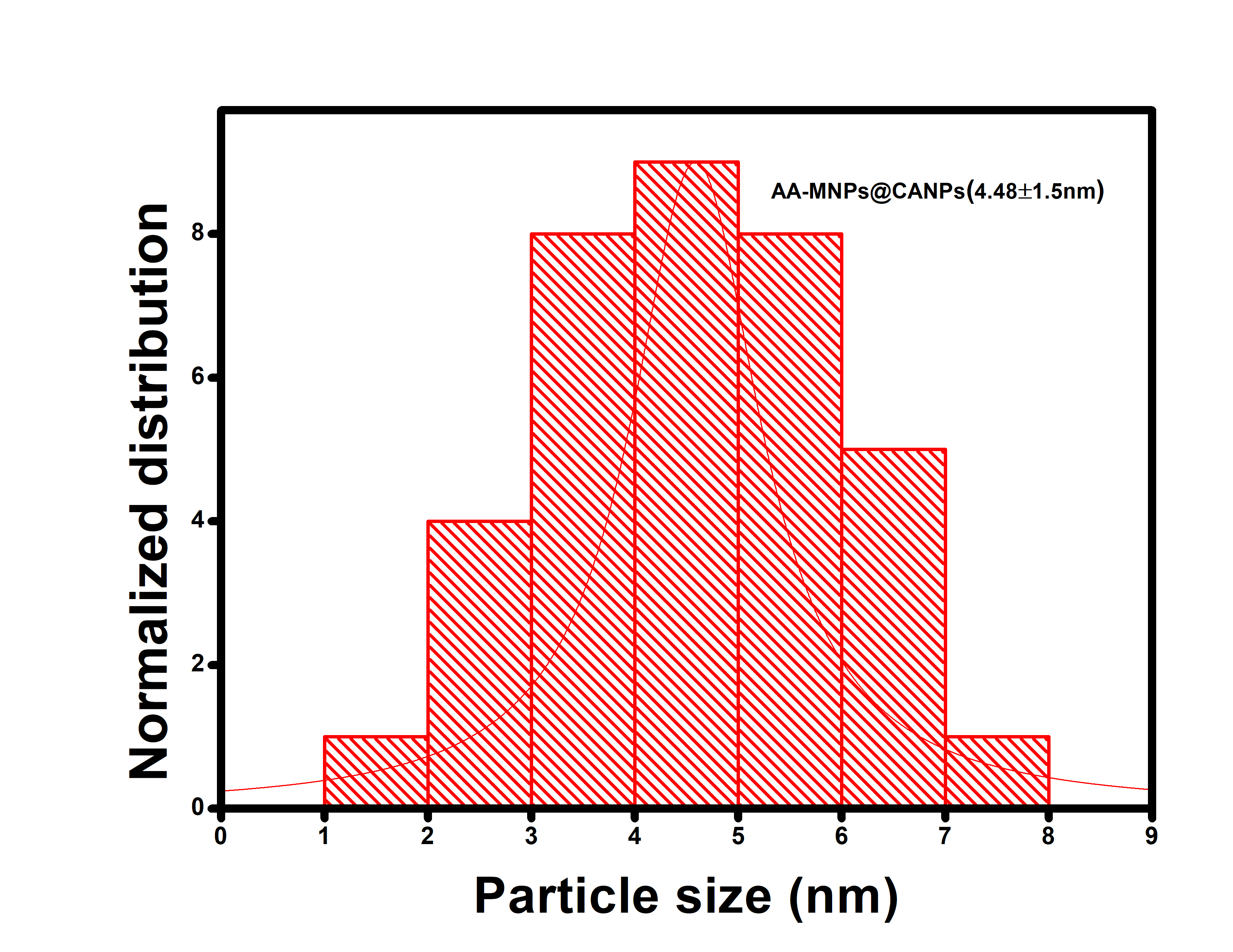

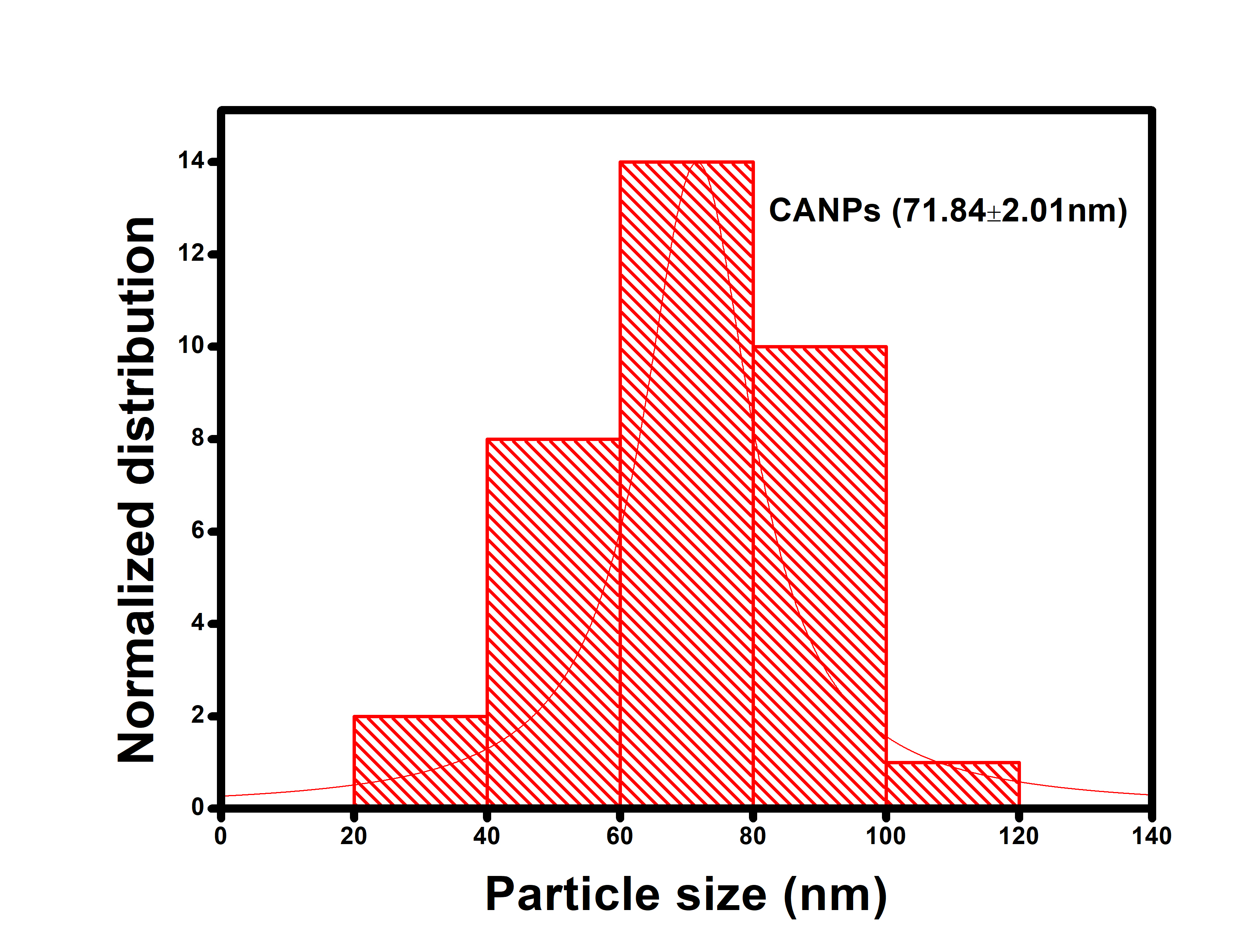


(A)

(B)

**Figure S1:** *Particle size distribution of nanomaterials (A) CANPs (B) AA-MNPs@CANPs.*

S2


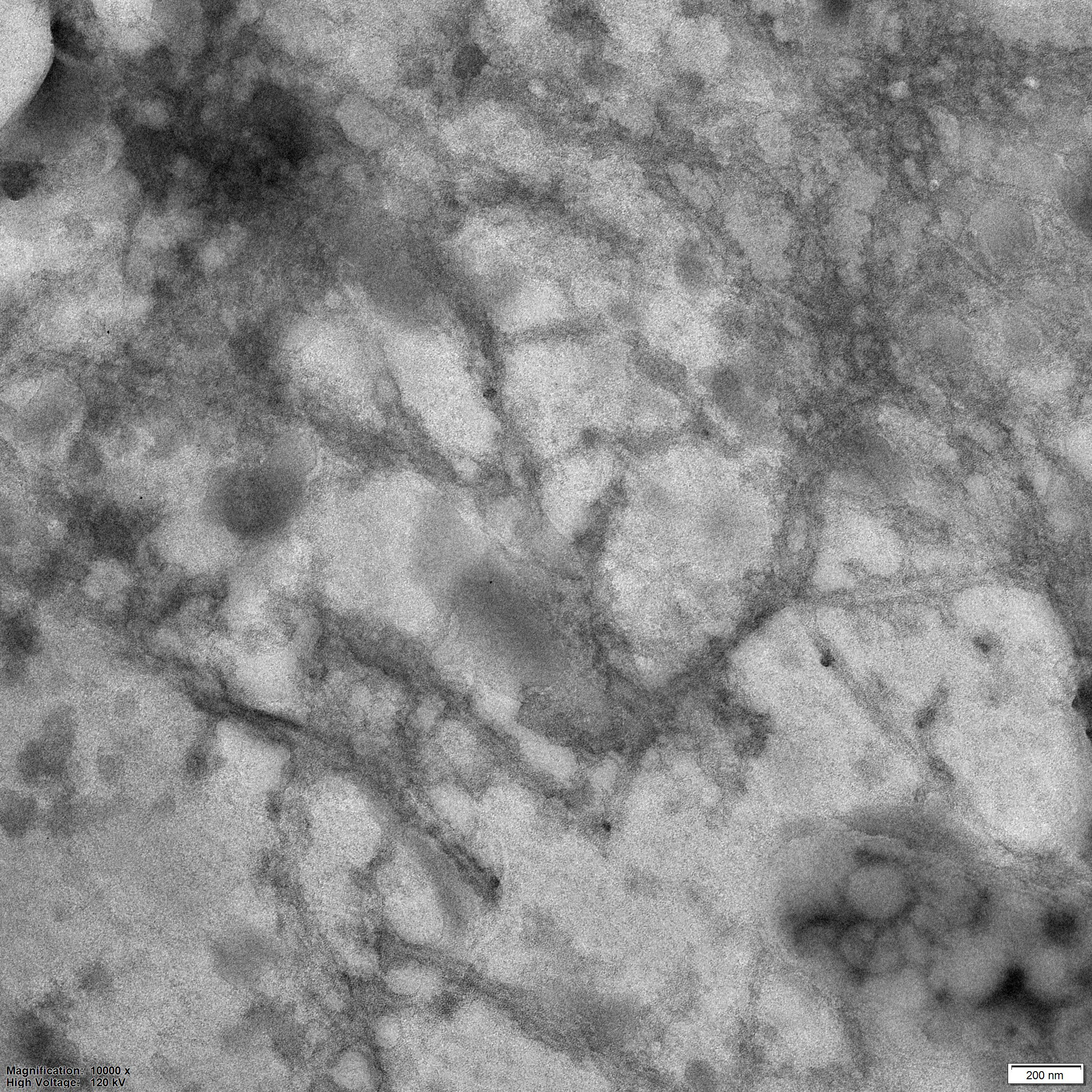

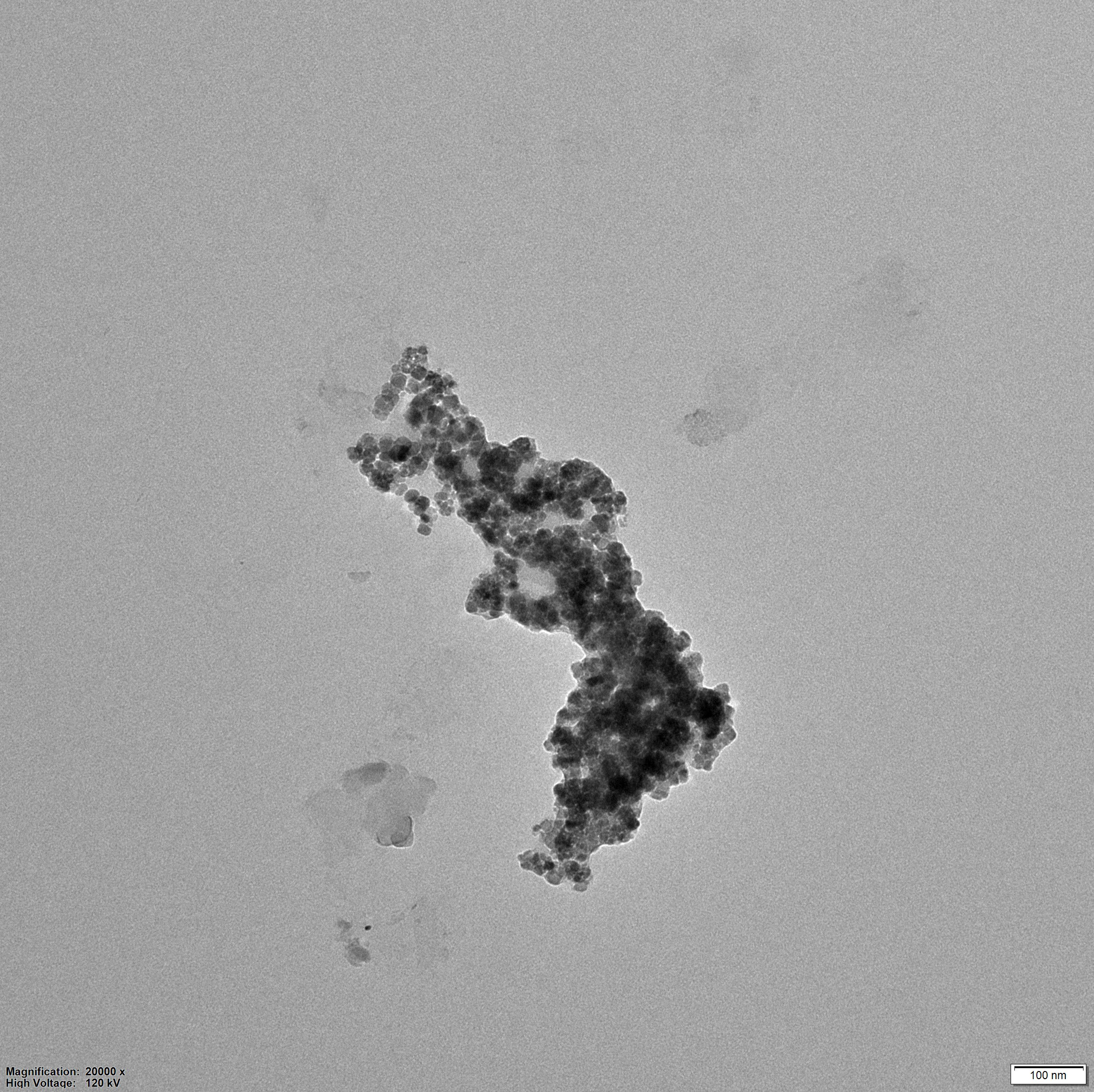


(B)

(A)

**Figure S2:** *TEM image of* *nanomaterials (A) CANPs (500 nm) (B) AA-MNPs@CANPs (200 nm).*


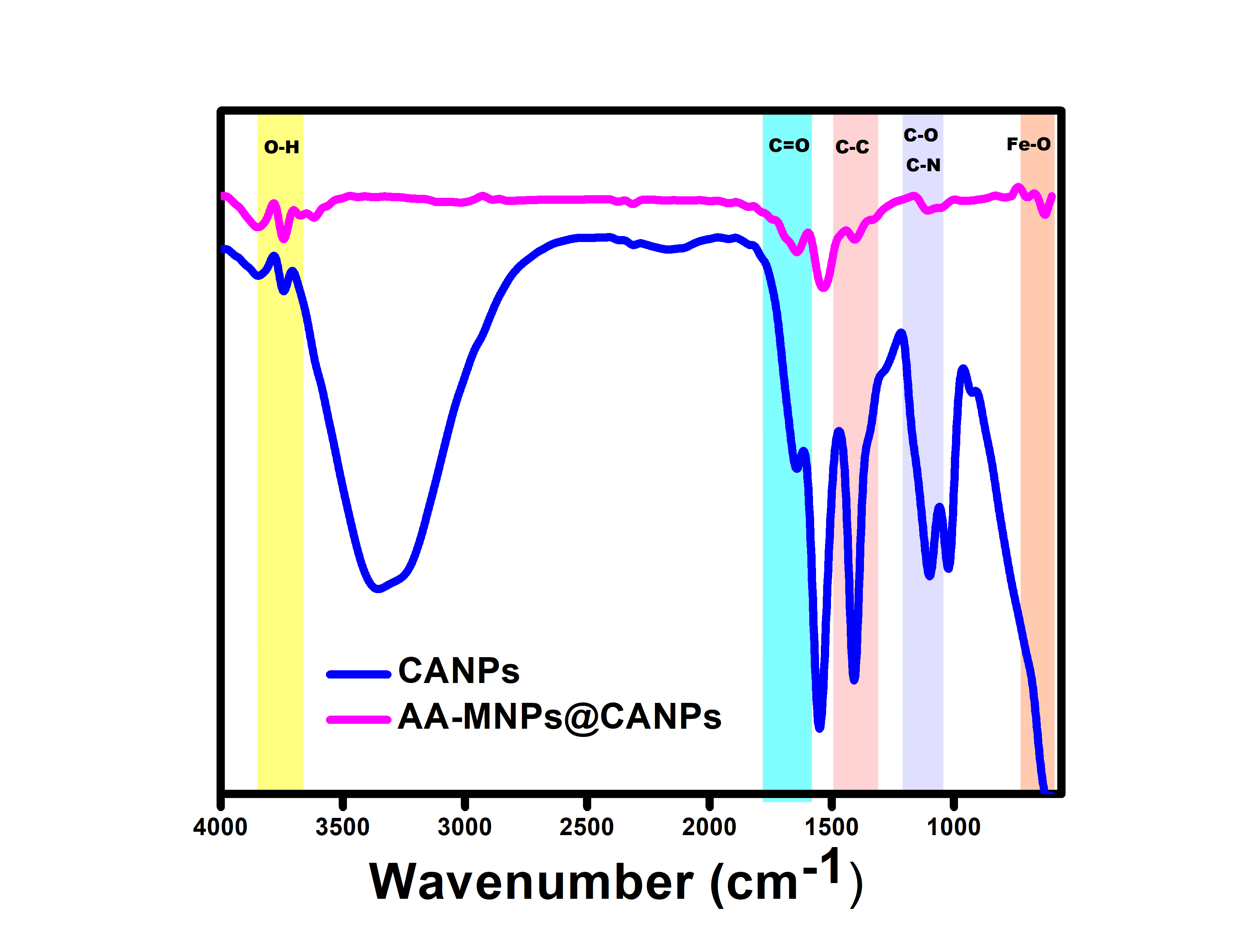

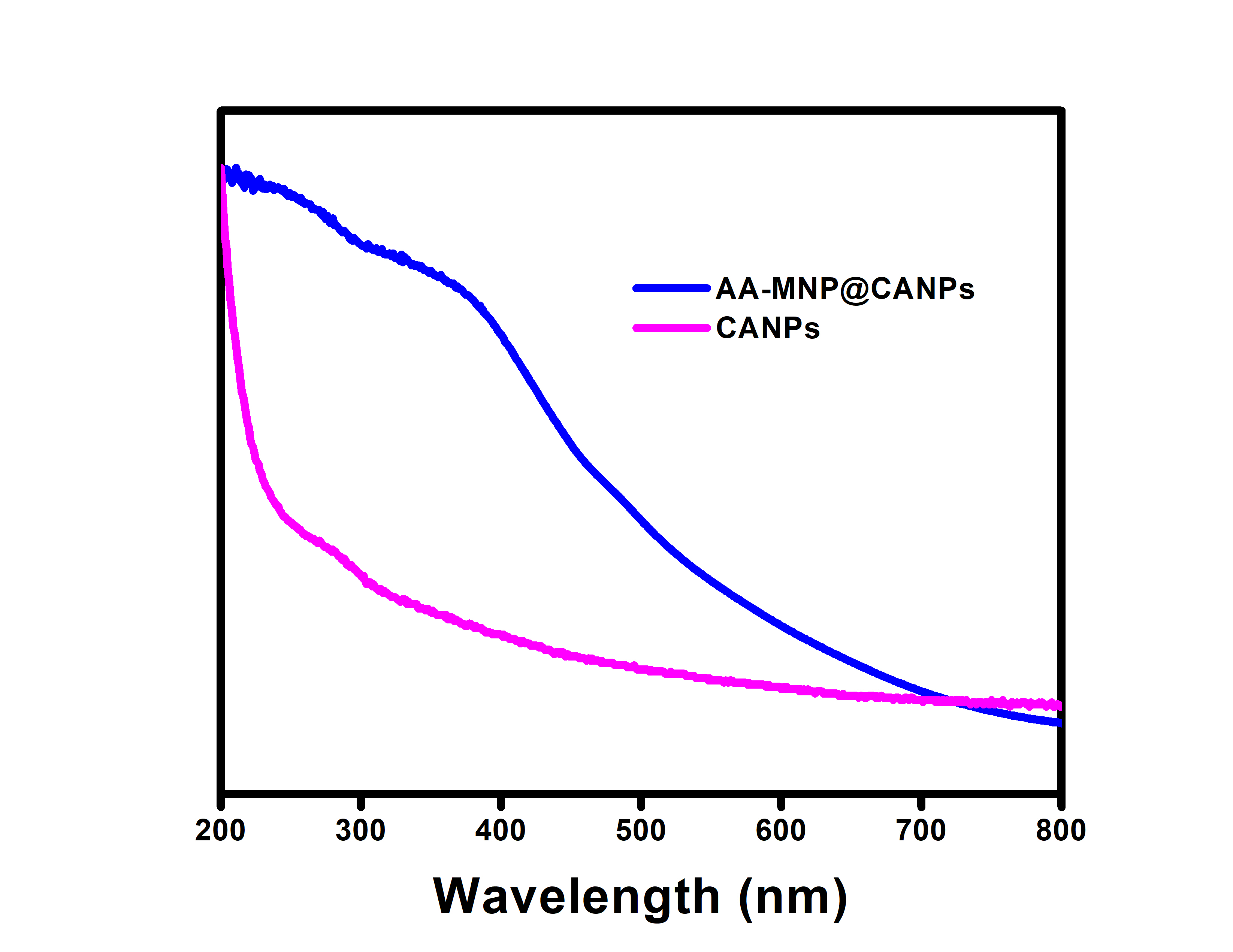


(B)

(A)

**Figure S3:** *(A) FT-IR spectra of CANPs and decorated nanomaterials (B) UV-VIS spectra of CANPs and AA-MNPs@CANPs.*

S3
